# Infectious triggers in IgA vasculitis in children: current evidence, causal attribution and immunopathogenic mechanisms

**DOI:** 10.1007/s00296-026-06295-x

**Published:** 2026-09-26

**Authors:** Filippos Filippatos, Dimitra Venieri, Evangelia Tsigkrou, Vasiliki Karava, Marianna Miliaraki, Konstantinos Kakleas

**Affiliations:** 1 First Department of Paediatrics, Medical School, National and Kapodistrian University of Athens, Aghia Sophia Children’s Hospital, Athens, 11527 Greece; 2 Second Department of Paediatrics, National and Kapodistrian University of Athens, P&A Kyriakou Children’s Hospital, Athens, 11527 Greece; 3 Pediatric Intensive Care Unit, University Hospital, Heraklion, Crete, 71003 Greece

**Keywords:** IgA vasculitis, Child, Infections, Immunoglobulin A, Immune complex diseases, Complement system proteins, Microbiota

## Abstract

IgA vasculitis (IgAV) is the most common systemic vasculitis in childhood. Its seasonal distribution and frequent respiratory or gastrointestinal prodromes implicate environmental exposures. However, microbiological detection temporally proximate to disease onset is insufficient to establish causality in an individual patient. To critically evaluate infectious exposures associated with pediatric IgAV, we distinguished epidemiological associations from incidental detection and infectious mimics, and separate direct IgAV evidence from mechanisms extrapolated from IgA nephropathy or general mucosal immunology. PubMed and MEDLINE were searched from inception through 6 August 2026 and supplemented by backward and forward citation tracking. Controlled-vocabulary and free-text terms addressed IgAV, pediatric populations, infection, specific pathogens, microbiota, vaccination, and immune mechanisms. The broad query retrieved 1,174 records before deduplication. Because screening was not prospectively logged, deduplicated screening and full-text counts could not be reconstructed; accordingly, no systematic-review or PRISMA completeness claim is made. Comparative evidence was most consistent for group A streptococcal exposure and population-level circulation of seasonal respiratory bacteria. Evidence for *Mycoplasma pneumoniae*, *Helicobacter pylori* in selected phenotypes, SARS-CoV-2, and specific enteric or parasitic infections was of lower certainty and was frequently case-based. Pediatric vaccine studies did not demonstrate a consistent excess risk. Evidence supports convergence of mucosal IgA induction, IgA1-containing immune complexes, complement activation, FcαRI-mediated myeloid signaling, neutrophil extracellular traps, and endothelial injury. Causal attribution requires integrated assessment of temporality, anatomical source, microbiological specificity, background exposure prevalence, and alternative diagnoses. Infectious investigations should be restricted to circumstances in which identification of a treatable infection, persistent antigen source, or immunosuppression-related hazard would alter management.

## Introduction and scope

IgA vasculitis (IgAV) is an IgA1-dominant small-vessel vasculitis characterized clinically by palpable purpura with articular, gastrointestinal, renal, or biopsy-supported vasculitic involvement [1, 2]. Pediatric classification criteria remain clinically relevant because they distinguish IgAV from thrombocytopenic purpura, septic embolic disease, urticarial vasculitis, Kawasaki disease, multisystem inflammatory syndrome in children, viral exanthems, drug eruptions, and other infectious mimics [2–4]. This distinction has direct therapeutic implications, because selected mimics require antimicrobial treatment or source control rather than escalation of immunosuppression.

The protein-homeostasis-system (PHS) hypothesis is considered only as an author-proposed conceptual model. It has neither been independently validated in IgAV nor supported by reproducible demonstration of an IgAV-specific pathogenic protein or peptide. Accordingly, it is treated as hypothesis-generating and does not influence evidence grades [5–9].

Previous publications have examined individual pathogens or restricted clinical settings, including SARS-CoV-2-related flares or incident IgAV [10–12], tuberculosis coexisting with IgAV [13], *Clostridioides difficile* infection [14], pandemic-era epidemiology [15], and gastrointestinal infection complicating established IgAV [16]. Most comprise adult cases, small case-based reviews, or narrow exposure contexts. In contrast, this review provides a pediatric, exposure-wide synthesis that distinguishes population-level association from patient-level causal attribution, applies a prespecified four-category interpretive framework, explicitly evaluates temporality, and separates direct IgAV evidence from evidence extrapolated from IgA nephropathy or general immunology.

Although IgAV has a marked pediatric predilection, adult-onset disease is well recognized. The main differences between pediatric and adult IgAV concern age distribution, incidence, comorbidity burden, renal-risk profile, and the immunological state of the host rather than entirely distinct immunopathogenic categories [17, 18]. Children have maturing immune systems, frequent mucosal viral and bacterial exposures, dynamic microbiota, and dense school-age transmission networks. Adults differ in cumulative exposure history, comorbidities, vascular reserve, and renal vulnerability [17, 18]. Accordingly, adult evidence is considered in this review only when it informs mechanisms, mimics, or rare infectious contexts and is not treated as equivalent to pediatric evidence.

Childhood IgAV is clinically heterogeneous. Many children present with mild, self-limited cutaneous, articular, or abdominal manifestations, whereas others develop severe gastrointestinal involvement, rapidly progressive vasculitis, relapse, or chronic IgAV nephritis [19–22]. Renal involvement is the principal determinant of long-term morbidity and is therefore central to pediatric nephrology guidance [22, 23]. Biomarker and therapeutic studies may be confounded when they pool patients with different ages, immune states, disease severities, organ phenotypes, treatment exposures, and intervals from disease onset. Thus, biomarkers measured during acute purpura, nephritic progression, post-corticosteroid treatment, or remission may represent distinct biological processes.

## Methods and evidence grading

### Search strategy and study selection

This narrative review was updated on 6 August 2026 in accordance with published recommendations for transparent narrative biomedical reviews [24]. PubMed and MEDLINE were searched from database inception through that date. The principal search combined (“IgA vasculitis” OR “Henoch-Schonlein purpura”) AND (child* OR pediatric*) AND (infection* OR trigger* OR pathogen* OR microbiota OR vaccin*). Supplementary searches addressed streptococci, *Mycoplasma pneumoniae*, *Helicobacter pylori*, SARS-CoV-2, respiratory viruses, enteric pathogens, parasites, complement, galactose-deficient IgA1, FcαRI, and neutrophil extracellular traps. The broad PubMed query retrieved 1,174 records before deduplication. Backward and forward citation tracking was performed for relevant guidelines, cohorts, reviews, and eligible case reports.

Eligible sources included pediatric epidemiological studies, comparative cohorts, reports with objective microbiological documentation, and mechanistic IgAV studies. Adult IgAV, IgA nephropathy, and general immunology studies were included only when pediatric IgAV evidence was insufficient and the source informed mechanism or differential diagnosis. Duplicate publications, non-IgA vasculitides, noninfectious exposures, unsupported secondary assertions, and reports lacking adequate phenotypic or microbiological detail were excluded. Evidence was prioritized according to pediatric relevance, objective microbiology, compatible temporality, and clinical utility. Because the original search was not prospectively logged, deduplicated screening and full-text counts could not be reconstructed; accordingly, this review is not presented as a systematic review and no PRISMA-based completeness claim is made.

### Evidence grading

The four-category (A–D) framework in Table 1 is an author-developed, nonvalidated interpretive tool informed by causal-inference principles and the Bradford Hill considerations [25]. It is distinct from GRADE and does not provide an independent determination of causality. Grades refer to the evidential support for each exposure–IgAV association rather than to the organism or biological plausibility alone. Patient-level attribution additionally requires compatible temporality and anatomical source, specific microbiological evidence, exclusion of alternative diagnoses, and consideration of background prevalence. Isolated seropositivity, remote IgG, common colonization, prolonged molecular shedding, and ecological synchrony were considered insufficient in isolation. The grades were developed during the narrative synthesis and represent the authors’ interpretive judgments based on the criteria in Table 1; they were not assigned through a blinded, independently duplicated systematic-review process.

**Table 1:** Author-developed framework for grading evidence supporting associations between infectious exposures and pediatric IgA vasculitis

Grade	Minimum evidence and attribution standard	Recommended terminology
A	Analytical comparative evidence, replicated pediatric series with objective microbiological documentation or a robust population-level association with compatible temporality and biological plausibility. Patient-level causal attribution nevertheless requires exposure-specific evidence.	Association supported by comparatively strong pediatric evidence; population-level trigger supported.
B	Multiple pediatric cases or small series with objective microbiological documentation and coherent temporality, or one methodologically robust pediatric series with adequate exclusion of major alternative diagnoses.	Probable association in selected patients; individual attribution requires case-specific evidence.
C	Isolated cases, adult-predominant or indirect pediatric evidence, inconsistent comparative findings or mechanistic support derived principally by analogy.	Possible association; patient-level causality not established.
D	No verified pediatric IgAV cases, theoretical mechanisms only, secondary citations without primary evidence,common infectious mimics or opportunistic complications or evidence favoring a non-IgA vasculitis.	No direct pediatric IgAV association established; evaluate principally as a mimic or complication.

*Note: The A-D framework was developed for this narrative synthesis and has not been externally validated. It standardizes terminology within the review and should not be interpreted as a formal certainty-of-evidence instrument.*

## Epidemiological and clinical context for infectious attribution

IgAV is the most prevalent systemic vasculitis of childhood and most frequently presents between 4 and 6 years of age, although incidence varies by geography, ethnicity, referral setting, and case definition [24–29]. This age distribution is compatible with interactions among common microbial exposures, age-dependent mucosal immune maturation, and the developing microbiota; it does not support attribution to a single predominant pathogen.

Large pediatric cohorts consistently identify palpable purpura as the defining manifestation, with articular, gastrointestinal, and renal involvement varying according to cohort composition, disease severity, and follow-up duration [19–22, 30]. Since renal findings may appear after the initial cutaneous eruption, absence of hematuria or proteinuria at presentation does not exclude subsequent IgAV nephritis [21–23].

Seasonality and antecedent infection histories are useful etiological signals, but they should be interpreted as hypothesis-generating rather than causality-proving observations [31, 32]. Older seasonal studies and contemporary time-series analyses support temporal clustering between childhood IgAV and respiratory pathogens, particularly respiratory bacterial circulation, but ecological associations cannot identify the responsible exposure in a specific child [31, 32].

Co-circulating viruses, bacterial carriage, school attendance, climate, health-care-seeking behavior, antibiotic exposure, vaccination coverage, and diagnostic intensity can all confound seasonal signals [25, 31–33]. Clinical phenotype can nevertheless suggest the most plausible anatomical source of antigenic stimulation when interpreted alongside objective testing [3, 4, 33].

A winter-spring presentation with tonsillitis, positive throat testing, or rising antistreptococcal titers is compatible with a pharyngeal IgA pathway [32, 34, 35]. Diarrhea, dysentery, mesenteric adenitis, or ulcer-like symptoms suggest gut antigen exposure, H. pylori-associated chronic mucosal stimulation, or an infectious mimic of IgAV enteritis [36–40]. Relapsing purpura with fever, pyuria, abscess, focal bone pain, a murmur, necrotic lesions, or persistently elevated inflammatory markers should prompt evaluation for a persistent antigen reservoir before the disease is labeled refractory autoimmunity [3, 4, 23, 33].

## Framework for temporality, causal attribution, and microbiota evidence

Infectious attribution in IgAV is difficult because many proposed agents are common childhood exposures, colonizers, or infections with prolonged serological or molecular footprints [4, 25, 32, 33]. Culture, antigen detection, or PCR/NAAT from a clinically relevant site during compatible symptoms supports active infection more strongly than a single delayed antibody result [34–36, 41, 42].

Serological tests such as ASO, anti-DNase B, *Mycoplasma* IgM, EBV serology, *H. pylori* serology, and SARS-CoV-2 antibodies answer different temporal questions and should not be interpreted as interchangeable proof of causation [34–36, 41, 42]. For this reason, the review separates preceding symptomatic illness from objective active infection at vasculitis onset, post-infectious immune disease, persistent antigen reservoirs, and incidental carriage or remote exposure [3, 4, 25, 32, 33].

Microbiota composition should be integrated into this framework because the nasopharynx, lower airway, intestine, skin, and urogenital tract contain microbial communities that vary with age, ethnicity, diet, hygiene, antibiotics, vaccination, geography, and household exposure [43–46]. Pediatric IgAV studies report oral and intestinal dysbiosis, but cross-sectional designs cannot determine whether dysbiosis is a cause, consequence, treatment effect, dietary effect, or marker of acute mucosal inflammation [43–46].

Cross-sectional microbiota studies cannot establish epithelial-barrier dysfunction or temporal directionality. Although barrier disruption could increase mucosal immune exposure to microbial products and damage-associated host molecules, this mechanism remains unconfirmed in pediatric IgAV [43–46]. The author-proposed PHS hypothesis posits that the biochemical characteristics and tissue distribution of released proteins, peptides, pathogen-associated molecular patterns, damage-associated molecular patterns, or related inflammatory products may be more relevant than taxonomic identity [5–9]. Within this review, the hypothesis is treated solely as an unvalidated conceptual model: no IgAV-specific biomarker or causal mechanism has been independently demonstrated, and PHS considerations do not affect evidence grading.

## Evidence synthesis by infectious exposure

Among bacterial exposures, group A beta-hemolytic Streptococcus has the most consistent supporting evidence. Pediatric cohort and case-control studies associate recent streptococcal exposure, pharyngitis, and elevated ASO or anti-DNase B titers with IgAV onset [34, 35]. A proposed mechanism is tonsillar epithelial and dendritic-cell stimulation by streptococcal cell-wall components and secreted antigens, with downstream induction of IL-1β, IL-6, TNF-α, CXCL8, BAFF/APRIL, T-follicular-helper-cell activity, and IgA1 plasmablast survival [34, 35, 47, 48]. This mechanism has not been established at the individual level, and ASO or anti-DNase B indicates recent exposure without specifying exact timing, anatomical source, or causal relevance [34, 35]. Pneumococci and other seasonal respiratory bacteria are supported principally by population-level associations; patient-level attribution requires a compatible respiratory syndrome and objective microbiological evidence because nasopharyngeal carriage is common [32, 33].

Evidence linking *Mycoplasma pneumoniae* to pediatric IgAV is limited to selected patients with compatible respiratory disease and objective microbiological evidence [41, 42]. PCR obtained during symptomatic illness or appropriately timed paired serology provides greater specificity than an isolated IgM result. Available reports remain case-level and do not establish a pathogen-specific TLR2/TLR6 cytokine pathway. Although Th17/IL-17 and T-follicular-helper-cell activation have been documented in pediatric IgAV [49, 50], these findings are not specific to *M. pneumoniae*.

*Helicobacter pylori* may function as a persistent mucosal antigen source in selected children with gastrointestinal-predominant, recurrent, or treatment-refractory IgAV, particularly in high-prevalence settings. Current evidence does not support a universal causal role [36, 37]. Chronic exposure to urease, CagA, VacA, lipopolysaccharide, and epithelial NF-κB signaling could maintain mucosal production of CXCL8, IL-1β, TNF-α, IL-6, IL-17 A, BAFF/APRIL, and IgA, thereby facilitating immune-complex formation [36, 37, 47, 48]. These mechanisms remain inferential.

Respiratory and systemic viruses are frequently reported before IgAV, but many reports rely on clinical history rather than objective testing and should be interpreted cautiously [51]. SARS-CoV-2 infection has also been reported before IgAV; however, attribution requires documented infection, compatible timing, a typical IgAV phenotype, platelet assessment, and exclusion of MIS-C or Kawasaki-like inflammation because background exposure is common [52–56].

Evidence linking vaccination to incident IgAV remains of low certainty. Two pediatric case reports described IgAV 4–12 days after COVID-19 vaccination but lacked source-population denominators [57, 58]. A case-crossover analysis of 167 children found no statistically significant association within 3 months (OR 1.6, 95% CI 0.8–3.0) and 1- and 2-month sensitivity analyses were also null [59]. In a case-control study of 288 cases and 617 controls, vaccination overall was not associated with IgAV. An imprecise MMR-specific association within 12 weeks was based on eight exposed cases (OR 3.4, 95% CI 1.2–10.0) [60]. A further case-crossover study of 193 children found no significant association within 1, 2, or 3 months (3-month OR 2.08, 95% CI 0.82–5.27) [61]. In a 2026 nationwide Norwegian cohort of 496,432 adolescents, 82.5% received a first mRNA-vaccine dose and no IgAV events occurred within prespecified 42-day risk windows after either dose. The rarity of the outcome nevertheless precluded exclusion of a small increase in risk [62]. No IgAV-specific causal window has been validated, and intervals of 42 days to 3 months should be interpreted as surveillance windows. Vaccination was therefore assigned Grade C, reflecting temporally compatible cases but inconsistent or null comparative evidence.

Enteric bacteria and parasites are mechanistically plausible exposures because intestinal inflammation may activate gut-associated lymphoid tissue, compromise epithelial barrier integrity, and promote antigen translocation, mucosal IgA production, complement activation, and neutrophil recruitment [38, 39, 63–65]. Associations with *Yersinia*, *Campylobacter*, hepatitis A virus, *Giardia*, *Entamoeba*, and norovirus are based predominantly on individual reports or case-based reviews; infection may precede IgAV, coexist at onset, or complicate established gastrointestinal disease [16, 38, 39, 63–65]. Where clinically indicated, targeted stool or hepatitis testing is required to distinguish infectious enterocolitis, hemolytic uremic syndrome, and IgAV-associated enteritis.

Fungal organisms are not established direct pediatric IgAV triggers and should usually be considered mimics or opportunistic complications, particularly when necrosis, immunocompromise, catheter exposure, or angioinvasion is suspected [4, 47, 48, 66, 67] (Table 2).

**Table 2:** Evidence-focused pathogen summary with immunological orientation

Exposure group	Evidence grade	Most defensible interpretation	Key immunological or methodological caution	References
Group A beta-hemolytic Streptococcus	A	Best-supported bacterial association, recent pharyngitis and streptococcal serology are frequent before IgAV.	ASO/anti-DNase B show recent exposure but not individual causality, pharyngeal IgA activation remains plausible but not pathogen-specific.	[34, 35]
Pneumococcus / seasonal respiratory bacteria	A	Population-level seasonal association between respiratory bacterial circulation and IgAV incidence.	Ecological signals cannot identify the causal organism in one child and carriage may be incidental.	[32, 33]
*Mycoplasma pneumoniae*	B	Association reported in selected children with compatible atypical pneumonia and objective microbiological evidence.	An isolated IgM result may persist or lack specificity, available reports do not establish a *Mycoplasma pneumoniae*-specific cytokine pathway.	[41, 42]
*Helicobacter pylori*	B	Possible persistent mucosal antigen reservoir in recurrent or gastrointestinal-dominant disease.	High carriage prevalence and serological limitations reduce specificity; active infection testing is preferred.	[36, 37]
SARS-CoV-2 infection	B	Reported contemporary association when timing, documentation, and classic IgAV phenotype fit.	Must distinguish IgAV from MIS-C, Kawasaki-like disease, viral exanthem, thrombocytopenia, and drug eruption.	[52–56]
Other respiratory viruses	C	Possible association with post-respiratory-illness IgAV when infection and temporality are objectively documented.	Common infections and prolonged PCR positivity limit patient-level inference.	[51]
Enteric bacteria and parasites	C	Case-based evidence supports possible antecedent exposures or complications of established gastrointestinal IgAV.	Infectious colitis, HUS, carriage, co-infection, and IgAV enteritis may confound attribution.	[38, 39]; [63–65]
Fungi	D	Primarily mimics, opportunistic complications, or theoretical immune cofactors.	Angioinvasive fungal disease requires infection-focused management rather than immunosuppression.	[4, 66]
Vaccines	C	Two pediatric COVID-19 vaccination case reports demonstrate temporal proximity, whereas comparative studies have not identified a statistically significant excess risk.	High background vaccination coverage, intercurrent infection, selection of risk windows, and reporting bias limit causal inference. Grade C reflects case-level temporal compatibility with inconsistent or null comparative evidence.	[57–62]

## Immunopathogenic framework

**Fig. 1:**
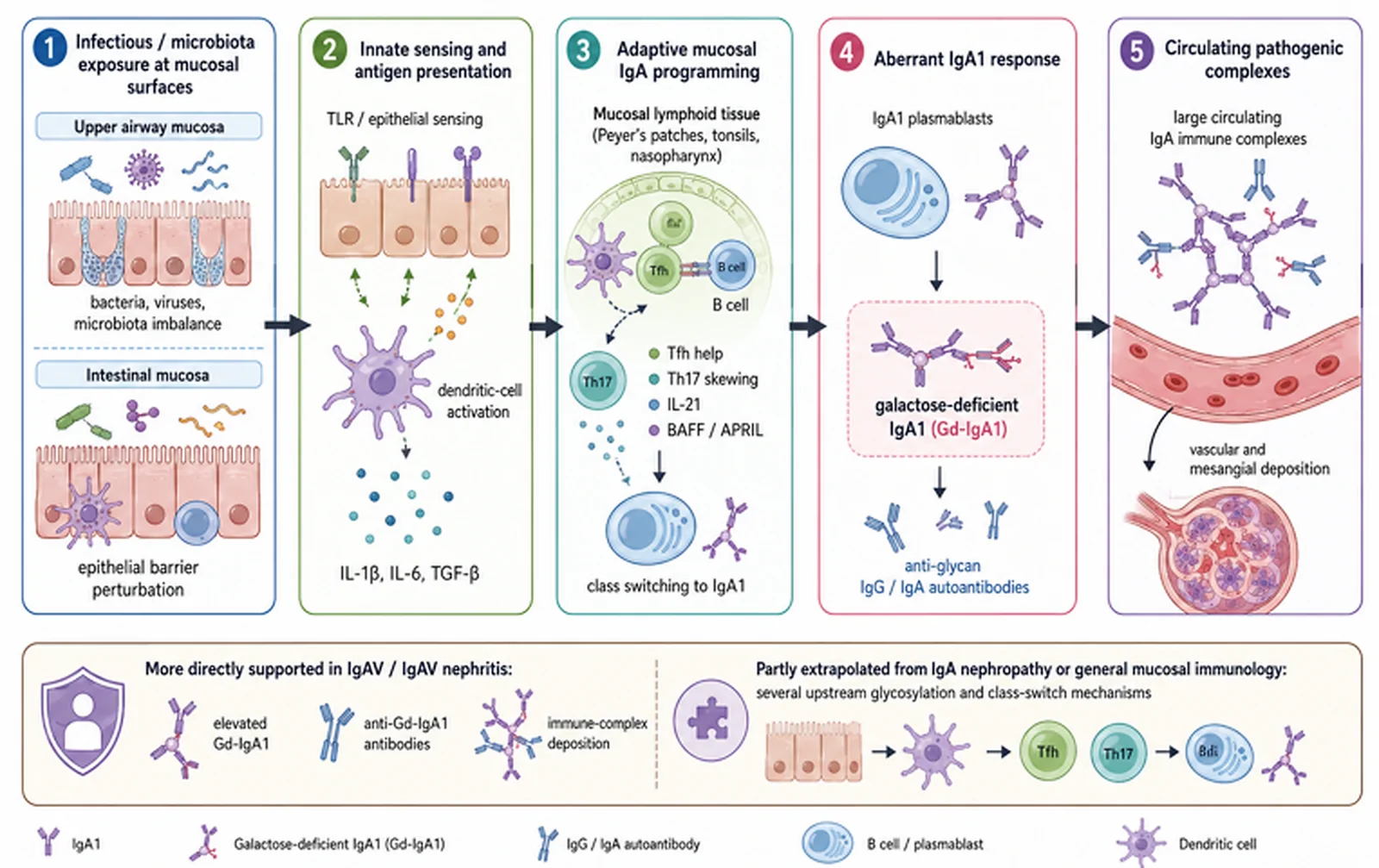
Upstream mucosal activation and IgA1 immune-complex formation. The model depicts respiratory or intestinal immune sensing, IgA class switching, galactose-deficient IgA1, anti-glycan antibodies, and circulating immune-complex formation

Pediatric IgAV is best framed as a threshold immune-complex disorder rather than a direct consequence of microbial invasion [66, 68–71]. The conventional multi-hit model begins with mucosal or systemic immune activation, increased IgA1 production, generation of galactose-deficient IgA1, formation of anti-glycan antibody-containing immune complexes, and deposition in susceptible small vessels or renal mesangium [66, 68–71]. This model is strongest for IgAV nephritis and should be applied more cautiously to isolated cutaneous, articular, or gastrointestinal IgAV because renal and non-renal lesions may not share identical antigenic or cellular drivers [66, 68–71] (Fig. 1).

Pattern-recognition signaling provides a plausible mechanistic link between infection and mucosal IgA responses. Epithelial cells and antigen-presenting cells detect microbial nucleic acids, lipoproteins, peptidoglycan, lipopolysaccharide, flagellin, and tissue-damage signals through Toll-like, RIG-I-like, NOD-like, and inflammasome-associated pathways [47, 48, 67]. The resulting cytokine milieu, including IL-1β, IL-6, TNF-α, CXCL8, type I/III interferons, BAFF, APRIL, TGF-β, IL-10, and IL-21, can promote IgA class switching, plasmablast survival, and systemic dissemination of mucosal humoral responses [47, 48, 67]. Direct demonstration of this sequence in pediatric IgAV remains limited.

In genetically or immunologically susceptible hosts, repeated mucosal stimulation may expand polymeric or aberrantly glycosylated IgA1-producing plasmablasts and promote formation of immune complexes containing anti-glycan IgG or IgA [70–74]. Increased galactose-deficient IgA1 (Gd-IgA1) and anti-Gd-IgA1 antibodies are directly supported in IgAV nephritis, whereas proposed regulation of glycosyltransferases is derived largely from IgA nephropathy and experimental B-cell systems [70–75]. Pediatric IgAV studies have identified circulating and PBMC microRNA profiles associated with disease activity, immunoglobulin concentrations, cytokines, and Th17/Treg balance, but have not demonstrated direct regulation of IgA1 glycosylation [76, 77]. The biological activity of Gd-IgA1-containing complexes is influenced by polymericity, glycan structure, antibody affinity, complex size, complement coating, clearance, and tissue access [70–74]. Following deposition, alternative- and lectin-pathway activation may amplify vascular and mesangial injury through C3b opsonization, C3a/C5a-mediated permeability and neutrophil priming, and terminal complement-complex formation [78–82]. Normal circulating C3 or C4 concentrations do not exclude local complement activation, while tissue, urine, and proteomic studies demonstrate alternative- and lectin-pathway signatures in IgAV [78–81] (Fig. 1).

**Fig. 2:**
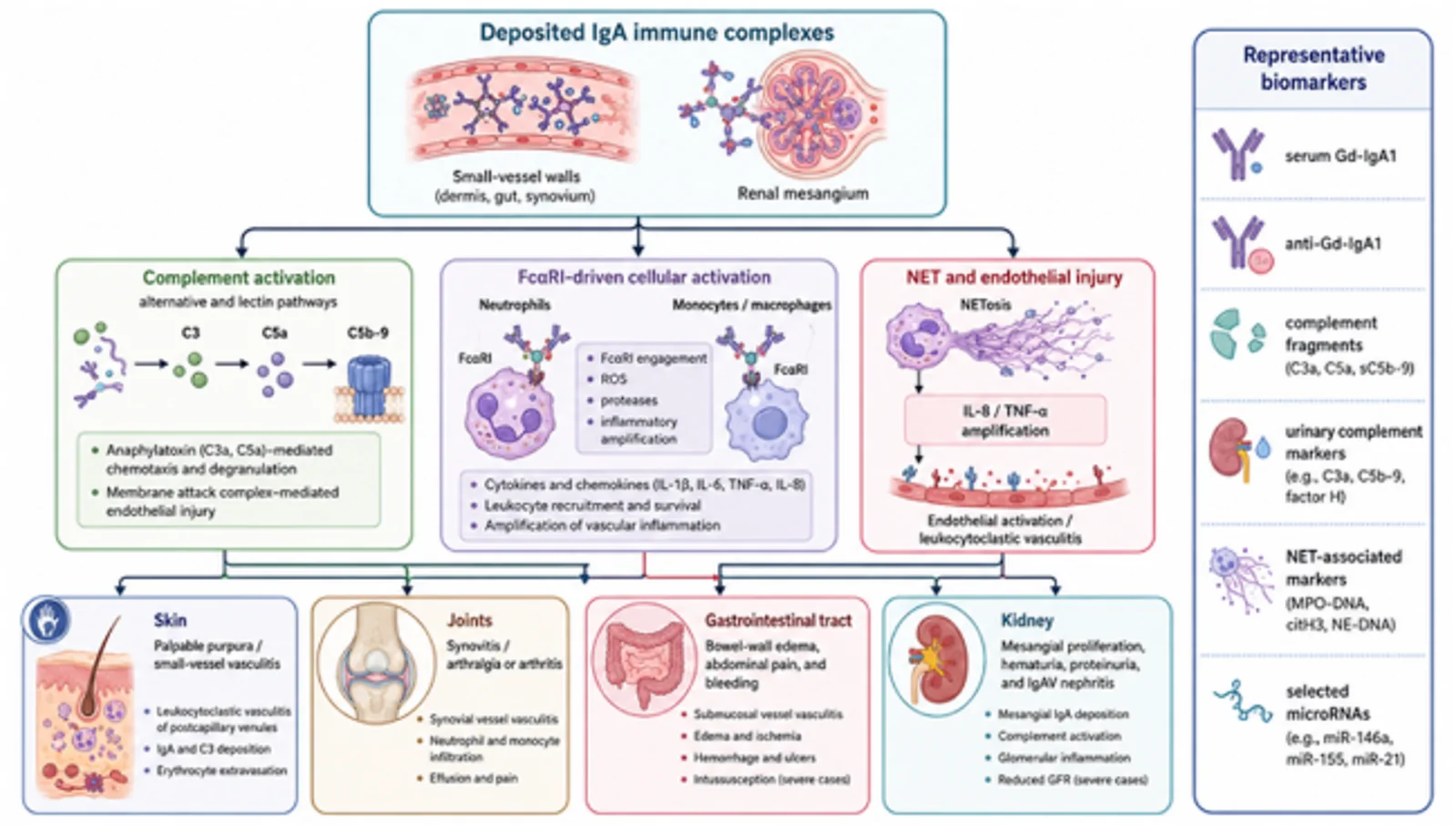
Downstream immune-complex effector pathways and organ manifestations. Based on deposited IgA-containing complexes, the model summarizes complement activation, FcαRI-mediated neutrophil and monocyte signaling, neutrophil extracellular-trap formation, endothelial injury, and cutaneous, articular, gastrointestinal, and renal phenotypes

Multivalent IgA complexes can cross-link FcαRI (CD89) on neutrophils and monocytes, activating Syk, PI3K, phospholipase Cγ, MAPK/ERK, calcium signaling, and NADPH oxidase, with consequent oxidative burst, protease release, and leukocytoclasia [83–85]. Neutrophil extracellular-trap formation may further amplify injury by retaining immune complexes and complement components within extracellular DNA-histone scaffolds and exposing endothelium to myeloperoxidase and elastase [83, 86, 87]. Endothelial activation, with increased adhesion molecules, permeability, von Willebrand factor release, and tissue-factor expression, provides a transition from circulating immune-complex abnormalities to tissue vasculitis [49, 50, 88–90]. Pediatric studies support IgA anti-endothelial-cell antibodies, IgA-induced endothelial CXCL8 production, Th17/Tfh dysregulation, and associations between cytokine profiles and organ involvement [49, 50, 88–90]. BAFF, APRIL, and BAFFR variants were not associated with IgAV susceptibility or phenotype in one study [91]. Epigenetic, HLA, single-cell, urinary Gd-IgA1, KM55, NET-related, and multi-omic studies remain exploratory and are not validated for routine exposure attribution or therapeutic selection [76, 77, 92–97] (Fig. 2).

Phenotypic heterogeneity may reflect variation in immune-complex size, polymericity, glycan structure, antibody affinity, complement coating, clearance, and tissue access [70–74]. The renal mesangium is subject to distinct hemodynamic and filtration conditions and expresses receptors including CD71, whereas dermal and gastrointestinal microvessels differ in endothelial activation, local complement regulation, Fc-receptor-bearing leukocytes, barrier permeability, and resident immune cells [66, 69, 78–90]. Deposition is therefore not a uniform biological endpoint, but vascular-bed characteristics, local inflammatory thresholds, host genetics, and exposure duration may contribute to cutaneous, gastrointestinal, articular, renal, or multisystem phenotypes. These mechanisms are biologically plausible but remain incompletely validated in paired pediatric tissues.

## Mechanisms of disease resolution and immune regulation

The self-limited course of most childhood IgAV suggests that resolution involves both removal of the initiating stimulus and active termination of inflammation. Plausible processes include clearance of infection or persistent mucosal antigen, restoration of epithelial barriers, reduced IgA immune-complex production, hepatic and phagocytic clearance, complement regulation, neutrophil apoptosis and efferocytosis, extracellular-trap degradation, and endothelial repair. Regulatory T- and B-cell responses, IL-10, TGF-β, and inhibitory Fc-receptor signaling may limit inflammatory amplification. However, increases in regulatory-cell populations during acute disease may represent compensation rather than effective resolution.

Direct pediatric evidence is limited. A prospective study comparing 30 children with acute IgAV, 30 in remission, and 40 healthy controls found higher regulatory T-cell proportions during acute disease and lower regulatory B-cell measures among patients with renal impairment [98]. These data support involvement of immunoregulatory pathways but do not establish mechanisms of spontaneous resolution. Longitudinal sampling from prodrome through convalescence is required to distinguish causal resolution pathways from secondary repair responses.

Treatment response in severe IgAV and IgAV nephritis principally reflects suppression of inflammatory amplification and organ injury and cannot be interpreted as evidence that an initiating infectious or antigenic exposure has been eliminated [3, 23, 99, 100]. Mechanistic studies should directly evaluate defined microbial or host antigens within circulating IgA complexes, renal deposits, and affected tissues rather than infer persistence from clinical course [66]. KM55 tissue staining and urinary Gd-IgA1 assays demonstrate approaches for detecting host-derived antigenic structures but do not identify microbial triggers [95, 96]. Table 3 summarizes direct IgAV evidence versus extrapolated or hypothetical mechanisms.

**Table 3:** Direct IgAV evidence versus extrapolated or hypothetical mechanisms

Domain	Directly supported in IgAV / IgAV nephritis	Extrapolated or hypothetical elements	Interpretive status in this review	References
Disease-defining deposits	IgA1-dominant vascular or renal deposits, C3 deposition, and leukocytoclastic neutrophilic vasculitis.	General immune-complex principles refine but do not define the phenotype.	Core IgAV biology.	[1, 66]; [68, 69]
Gd-IgA1 / anti-Gd-IgA1	Increased Gd-IgA1 and anti-Gd-IgA1 antibodies, particularly in IgAV nephritis.	Assay thresholds, complex stoichiometry, glycosyltransferase regulation, and prognostic cutoffs are mostly IgAN-derived.	Central renal pathway, not universal diagnostic proof.	[70, 71]; [72–75]; [95, 96]
Mucosal IgA induction	Pediatric IgAV studies report associations with respiratory or gastrointestinal exposures and Tfh/Th17 dysregulation; one genetic study found no association involving BAFF/APRIL pathway variants.	Precise mucosal-homing lineage tracing and enzyme control are mainly IgAN/general biology.	Mechanistic bridge stated cautiously.	[32, 33]; [47– [50, 67]; [91]
Complement	Alternative and lectin activation in skin, kidney, urine, and proteomic studies.	Evidence for complement-directed treatment in IgAV is limited and is derived principally from IgA nephropathy or general complement biology.	Potential endotype, not routine treatment indication.	[78– [82, 101]
Neutrophils / NETs / FcαRI	Neutrophilic histology and NET signatures in human and animal IgAV studies.	Receptor-level FcαRI signaling is partly extrapolated from general IgA biology.	Effector mechanism with translational gaps.	[83–87]; [97]
Endothelium	IgA-AECA and IgA-induced endothelial IL-8 responses are reported in pediatric IgAV.	Specific target antigens and initiating role remain unresolved.	Amplifier or biomarker unless causality is demonstrated.	[88, 89]
Microbiota	Oral and gut dysbiosis are associated with childhood IgAV.	Directionality, strain-level causality, and treatment implications remain unproven.	Contextual susceptibility factor.	[43–46]
Resolution and immune regulation	Prospective pediatric data demonstrate differences in regulatory T- and B-cell measures across acute disease, remission, and renal phenotypes.	Antigen and immune-complex clearance, complement regulation, neutrophil efferocytosis, extracellular-trap degradation, and endothelial repair are supported principally by extrapolated evidence.	Biologically plausible resolution pathways; direct longitudinal pediatric evidence remains limited.	[47, 48, 67, 82, 84, 85, 98, 101]

## Prognostic implications of infectious exposures

Available evidence does not yet demonstrate that pathogen identity independently determines severity, relapse, organ involvement, or renal outcome in pediatric IgAV after adjustment for age, baseline phenotype, disease duration, host susceptibility, treatment, and sampling bias [19–23].

A child with streptococcal-associated, *Mycoplasma*-associated, post-viral, or SARS-CoV-2-associated IgAV may develop either mild or severe disease depending on the host threshold, immune-complex burden, and organ-specific effector injury [34, 41, 52, 66, 78].

The most reproducible prognostic signals remain clinical and nephrological, including renal involvement, persistent proteinuria, nephritic or nephrotic features, gastrointestinal severity, recurrence, older age in selected cohorts, and biopsy findings when renal biopsy is indicated [19–23]. Infectious source may still have prognostic relevance when it represents a persistent antigen reservoir, abscess, urinary focus, endocarditis, chronic gastric infection, tuberculosis, strongyloidiasis, or invasive fungal mimic that alters the safety of immunosuppression [3, 4, 23, 33, 99]. The clinically actionable question is therefore whether a treatable or hazardous infection is present and whether its identification changes antimicrobial therapy, source control, immunosuppression timing, or renal surveillance [3, 4, 23, 33, 99].

## Clinical implications

Routine broad infectious screening is not indicated for most children with typical IgAV because indiscriminate testing increases incidental positives and rarely changes management [3, 4, 23, 99]. Baseline assessment should establish the IgAV phenotype, exclude thrombocytopenia or sepsis, and define organ severity using blood pressure, complete blood count with platelet count, urinalysis, urine protein/creatinine ratio, serum creatinine, and focused evaluation of gastrointestinal or renal disease [3, 4, 23, 99].

Infectious testing should be targeted to scenarios in which the result affects antimicrobial therapy, infection-control measures, safety of immunosuppression, or differential diagnosis [3, 4, 23, 33, 99]. Antibiotic or antiparasitic therapy should be used for documented infections according to standard pediatric guidance rather than as nonspecific therapy for IgAV itself [3, 4, 23, 99]. Corticosteroids, immunosuppressive agents, anti-inflammatory drugs, and biologics may control overactive or incomplete immune responses, but they should not be described as eliminating the etiological substance unless an infectious focus is also controlled [3, 5, 23, 99, 100]. Severe renal or gastrointestinal disease requires timely organ-directed therapy, and unnecessary delay of immunomodulation solely because an antecedent infection is suspected may be harmful [3, 23, 99].

A pragmatic clinical approach is to recognize and treat severe IgAV promptly while evaluating for active infection, persistent antigen reservoirs, or infectious mimics when fever, focal symptoms, recurrence, necrosis, epidemiological exposure, or planned high-dose immunosuppression makes these possibilities clinically plausible [3, 4, 23, 33, 99].

## Research priorities

Future studies should prioritize temporality, clinical phenotype, and causal attribution rather than compile undifferentiated pathogen lists. Informative designs would enroll children at IgAV onset, document infections systematically by anatomical source, collect acute and convalescent microbiological samples, record antibiotic and vaccine exposures, and prospectively assess renal, gastrointestinal, articular, and relapse outcomes. Analyses should be stratified by age, sex, ethnicity, season, disease severity, interval from prodrome to purpura, treatment exposure, and renal phenotype.

Pathophysiological investigations should determine whether defined microbial antigens or host-derived molecules are detectable within IgA-containing complexes, renal deposits, or affected tissues. Longitudinal integration of microbiome sequencing, IgA1 glycomics, B-cell receptor sequencing, anti-Gd-IgA1 specificity, complement and NET biomarkers, endothelial assays, single-cell transcriptomics, and regulatory-cell phenotyping may distinguish antecedent exposures from inflammatory amplification and tissue repair. Predictions derived from the PHS hypothesis require preregistration, operationally defined outcomes, and independent evaluation by investigators not involved in its formulation.

## Conclusion

Current evidence supports a convergent immune-complex model of infection-associated pediatric IgAV rather than distinct pathogen-specific disease entities. Microbial exposures may interact with age-dependent host susceptibility, mucosal IgA responses, IgA1-containing immune complexes, complement activation, neutrophil effector pathways, extracellular traps, and vascular-bed-specific biology to influence phenotype. Serological positivity, colonization, ecological seasonality, and isolated case reports are insufficient for individual causal attribution. Infectious investigations should therefore be directed toward treatable or management-relevant conditions while standard renal surveillance is maintained. The PHS hypothesis remains an author-proposed, unvalidated conceptual model and is not incorporated into causal evidence grading.

## Declarations

### Originality and responsibility statement

This manuscript has not been copied from or published in another source.
